# Engineering mediator-based electroactivity in the obligate aerobic bacterium *Pseudomonas putida* KT2440

**DOI:** 10.3389/fmicb.2015.00284

**Published:** 2015-04-10

**Authors:** Simone Schmitz, Salome Nies, Nick Wierckx, Lars M. Blank, Miriam A. Rosenbaum

**Affiliations:** Aachen Biology and Biotechnology, Institute of Applied Microbiology, RWTH Aachen UniversityAachen, Germany

**Keywords:** *Pseudomonas putida*, phenazines, mediated electron transfer, microbial electrocatalysis, bioelectrochemical system, oxygen limitation

## Abstract

*Pseudomonas putida* strains are being developed as microbial production hosts for production of a range of amphiphilic and hydrophobic biochemicals. *P. putida*'s obligate aerobic growth thereby can be an economical and technical challenge because it requires constant rigorous aeration and often causes reactor foaming. Here, we engineered a strain of *P. putida* KT2440 that can produce phenazine redox-mediators from *Pseudomonas aeruginosa* to allow partial redox balancing with an electrode under oxygen-limited conditions. *P. aeruginosa* is known to employ its phenazine-type redox mediators for electron exchange with an anode in bioelectrochemical systems (BES). We transferred the seven core phenazine biosynthesis genes *phzA-G* and the two specific genes *phzM* and *phzS* required for pyocyanin synthesis from *P. aeruginosa* on two inducible plasmids into *P. putida* KT2440. The best clone, *P. putida* pPhz, produced 45 mg/L pyocyanin over 25 h of growth, which was visible as blue color formation and is comparable to the pyocyanin production of *P. aeruginosa*. This new strain was then characterized under different oxygen-limited conditions with electrochemical redox control and changes in central energy metabolism were evaluated in comparison to the unmodified *P. putida* KT2440. In the new strain, phenazine synthesis with supernatant concentrations up to 33 μg/mL correlated linearly with the ability to discharge electrons to an anode, whereby phenazine-1-carboxylic acid served as the dominating redox mediator. *P. putida* pPhz sustained strongly oxygen-limited metabolism for up to 2 weeks at up to 12 μA/cm^2^ anodic current density. Together, this work lays a foundation for future oxygen-limited biocatalysis with *P. putida* strains.

## Introduction

*Pseudomonas putida* and closely related species such as *Pseudomonas* sp. VLB120 are gaining increasing scientific and biotechnological interest because of their comparatively high tolerance toward amphiphilic and hydrophobic chemicals (Vickers et al., 2010; Lang et al., 2014; Nikel et al., 2014). This property allows these strains to grow in the presence of or even produce otherwise toxic chemicals like aromatic phenols and styrenes (Wierckx et al., 2005; Verhoef et al., 2009; Volmer et al., 2014). Furthermore, biocatalysis in biphasic aqueous/organic systems allows direct product removal of hydrophobic biochemicals and could simplify biotechnological processes (Blank et al., 2008; Heerema et al., 2011; Ütkür et al., 2012; Volmer et al., 2014). On the other hand, during the recombinant production of, e.g., valuable biodetergents like rhamnolipids (Wittgens et al., 2011), the required aeration leads to strong reactor foaming, which is technically hard to handle with conventional antifoam technologies (Küpper et al., 2013). In this case, the organism's obligate aerobic nature poses a severe challenge for reactor and process engineering.

One way to limit the aeration rate and thus, problems with reactor foaming would be to use a strain of *P. putida* that could produce rhamnolipids under anaerobic or oxygen limited conditions. Naturally, *P. putida* is obligately aerobic and no fermentative or alternative respirative energetic pathways are known. Attempts to engineer nitrate respiring recombinant strains of *P. putida* showed some success (Steen et al., 2013). The engineered strain was able to sustain metabolic activity under anaerobic conditions with nitrate as alternative electron acceptor, but was not able to grow under these conditions. Another approach tried to establish ethanol fermentation for anaerobic redox balancing, with similar results (Nikel and de Lorenzo, 2013).

Our research targets a different approach for metabolic redox balancing under oxygen-limited conditions via electrochemical discharge of surplus metabolic reducing equivalents. The addition of synthetic redox mediators like neutral red or methylene blue, as electron shuttles between a microbial cell and an anode as electron acceptor, was already explored at the end of the last century (Park et al., 1999). However, low efficiencies, costly and non-sustainable mediator addition, and possible environmental toxicity of the synthetic mediators prevented these scientific explorations from moving to technical applications.

Recent discoveries and investigations of natural microbial electron discharge to extracellular anodes might open new strategies for bioelectrochemical production processes (Rosenbaum and Henrich, 2014). Natural electron exchange between microorganisms and electrodes can be differentiated into direct and indirect or mediated electron transfer (MET). Direct, redox protein-based (e.g., via *c*-type cytochromes) electron transfer, for example, is exhibited by dissimilatory metal reducing bacteria like *Shewanella oneidensis* (Bretschger et al., 2007; Rosenbaum et al., 2012) or *Geobacter sulfurreducens* (Bond and Lovley, 2003; Lovley, 2008; Lovley et al., 2011). A model organism for natural MET is *Pseudomonas aeruginosa* (Rabaey et al., 2005; Venkataraman et al., 2010, 2011), which synthesizes and secretes a collection of phenazine-type redox molecules with diverse ecological and energetic functions.

The first scientific studies to utilize direct electron transfer to an anode for non-native metabolic redox balancing have recently been reported. For example the heterologous conversion of glycerol to ethanol was enabled through stoichiometric redox balancing with an anode in *S. oneidensis* (Flynn et al., 2010). The basic enzymatic elements of extracellular electron discharge from *S. oneidensis* have been heterologously expressed in *Escherichia coli* providing a first ability for direct extracellular electron discharge to this important biotechnological production host (Jensen et al., 2010; Goldbeck et al., 2013). In an update of this work, the engineered *E. coli* was able to produce more oxidized products by discharging electrons to an anode (TerAvest et al., 2014). However, any metabolic redox balancing via direct electron transfer requires a two dimensional biofilm-based production process on an electrode, which might limit volumetric reaction rates and yields. The alternative of intrinsic mediated electron discharge, which is not directly coupled to an electrode surface, circumvents this drawback. In a previous study, the biosynthesis of flavin redox mediators was enhanced in *S. oneidensis*, which also uses intrinsic redox mediators for extracellular electron exchange, and could increase microbial current and power production (Yang et al., 2015). In this work, we developed and investigated recombinant mediator-based redox balancing in oxygen limited *P. putida*.

In the MET model organism *P. aeruginosa*, phenazine synthesis is tightly controlled by the cell-to-cell communication quorum sensing system, and also by environmental factors like oxygen or iron availability (Sakhtah et al., 2014). The synthesis is based on chorismate as a precursor and proceeds via the core phenazine molecule phenazine-1-carboxylic acid (PCA) to fan out to the derivatives pyocyanin (PYO), 1-hydroxyphenazine (1-OHPHZ), and phenazine carboxamide (PCN), each with different redox properties (Wang and Newman, 2008; Mentel et al., 2009). Among these, PYO is considered as the most important and redox active phenazine (Venkataraman et al., 2011). Phenazines first became known as antimicrobial compounds because they generate reactive oxygen species (e.g., superoxide) and therefore rank among *P. aeruginosa*'s many pathogenicity factors. *P. aeruginosa* is able to resist their toxicity by producing superoxide dismutase and catalase. Later it was discovered that *P. aeruginosa* phenazines also affect iron bioavailability, enhance biofilm formation, oxidize the intracellular NAD(P)(H) pool, and enable anaerobic survival via the shuttling of metabolic electrons to far-away electron acceptors (Hernandez et al., 2004; Pierson and Pierson, 2010; Wang et al., 2010; Glasser et al., 2014). Examples of such alternative electron acceptors are solid mineralic compounds in the environment (Wang et al., 2011) or an anode in bioelectrochemical systems (BES) (Venkataraman et al., 2010; Wang et al., 2010).

Phenazine synthesis to PCA in *P. aeruginosa* is encoded by two almost identical 7-gene operons *phzA1-G1* and *phzA2-G2* (Mentel et al., 2009). For the synthesis of the most dominant phenazine PYO, two further genes *phzM* and *phzS*, encoding a methyltransferase and a monooxygenase respectively, are required. Here, we heterologously expressed the 7-gene cluster *phzA1-G1* and *phzM*+*phzS* from *P. aeruginosa* PAO1 on two compatible plasmids in the non-pathogenic *P. putida* KT2440. We show a first successful proof-of-principle for oxygen-limited redox balancing of *P. putida* with an anode as alternative electron acceptor and provide some quantitative insight into metabolic and energetic changes of this new phenazine producing *P. putida* strain. Altogether, this work forms a foundation for further engineering of solvent tolerant *P. putida* for oxygen-limited, electrochemically steered biocatalysis.

## Materials and methods

### Strains and media

*P. putida* strain KT2440 (DSM 6125, ATCC 47054) was used as the host for recombinant expression of phenazine synthesis genes. *E. coli* DH5α (New England Biolabs) was used for intermediary cloning steps. For strain maintenance and cloning experiments, both cultures were grown in LB medium with or without antibiotics as required at 30°C (*P. putida*) or 37°C (*E. coli*) and shaken at 200 rpm. For *P. putida* characterization and bioelectrochemical testing, strains were cultivated at 30°C in mineral salt medium with a final composition (per L) of 5 g glucose (27 mM), 3.88 g K_2_HPO_4_ (22 mM), 1.63 g NaH_2_PO_4_ (14 mM), 2.00 g (NH_4_)_2_SO_4_, 0.1 g MgCl_2_x6H_2_O, 10 mg EDTA, 2 mg ZnSO_4_x7H_2_O, 1 mg CaCl_2_x2H_2_O, 5 mg FeSO_4_x7H_2_O, 0.2 mg Na_2_MoO_4_x2H_2_O, 0.2 mg CuSO_4_x5H_2_O, 0.4 mg CoCl_2_x6H_2_O, 1 mg MnCl_2_x2H_2_O, and antibiotics as required (Hartmans et al., 1989).

### Genetic engineering of *P. putida* for phenazine synthesis

Descriptions and sources for all strains, plasmids, and primers used in this study are given in Supplementary Tables S1, S2. Genes *phzA1-G1*, *phzM*, and *phzS* were amplified from *P. aeruginosa* PAO1 genomic DNA. Plasmid construction for pBNTphzA-G and pJNNphzMS (Figures S1A,B) was planned and performed according to the New England Biolabs NEBuilder online tool for Gibson Assembly (Gibson et al., 2009; NEBuilder, 2013). Gibson cloning fragments were generated by PCR using Precisor High-Fidelity DNA Polymerase (BioCat GmbH, Heidelberg) with the primers from Table S2, followed by assembly and transformation of pBNTphzA-G and pJNNphzMS individually into *E. coli* DH5α with the NEB Gibson Assembly Kit. Transformants were selected on LB agar plates with kanamycin (Km, 50 μg/mL) or gentamycin (Gm, 30 μg/mL), respectively. The constructs were verified by PCR, restriction analysis and full-length sequencing using primers 7–16 (GATC, Germany). Thereafter, pBNTphzA-G and pJNNphzMS were co-transformed into *P. putida* KT2440 via electroporation as described in reference (Choi et al., 2006). Five colonies from Km + Gm plates were tested and confirmed for both plasmids via colony PCR and restriction digest (as above). For functional testing, these five clones were then inoculated into 50 mL LB medium containing antibiotics at a starting OD = 0.1 and induced with 0.1 mM (16 μg/mL) salicylate for gene expression and phenazine synthesis. A blue color, typical for pyocyanin synthesis, was observed after ~6 h and permanent glycerol stocks of the *P. putida* pPhz strains were prepared.

### Bioelectrochemical systems

### Setup of bioelectrochemical systems

Three identical bioelectrochemical reactors consisted of water-jacketed glass vessels (custom made, Gassner Glastechnik, Munich) and a three electrode setup: a working electrode (anode)—7.3 × 4.7 × 0.9 cm carbon comb (geometrical surface area = 156.32 cm^2^) attached to a graphite rod, a counter electrode—7.3 × 2.2 × 0.9 cm carbon block (49.22 cm^2^) attached to a graphite rod and a reference electrode RE—Ag/AgCl, saturated KCl (192 mV vs. SHE at 30°C). All port installations for electrodes, gas in and gas out as well as sampling were sealed with butyl rubber gaskets or septa. Reactors were operated with 500 mL culture volume, temperature controlled at 30°C with a recirculating water bath and stirred at 200 rpm with a magnetic stir bar.

### Operation of bioelectrochemical systems

Reactors were potentiostatically controlled by a potentiostat (VMP3, BioLogic) at 0.2 V vs. RE and the current reply of the microbial culture were recorded after 24 h of blank media measurements. Microbial experiments were performed under four different oxygen-limited conditions as described in detail in Table 1. For passive aeration, the headspace of the reactors was open to the atmosphere via a 0.2 μm sterile PTFE vent filter (Millipore, Billerica, MA). For active aeration, the gas inlet port was connected to an aquarium air pump via a sterile vent filter at a flow rate of 40 mL/s for headspace aeration in oxygen level II and 0.5 mL/s for media aeration in oxygen level III/III-. Dissolved oxygen concentrations for the different aeration levels were measured with optical oxygen sensors (Hamilton, incl. Hamilton Device Manager Software, Switzerland). Before studying the engineered *P. putida* pPhz strain in depth, we also investigated unmodified *P. putida* KT2440 under passive headspace aeration with the artificial addition of 5, 15, or 25 μg/mL PYO (Sigma-Aldrich, St. Louis, MO).

**Table 1:** **Oxygen limited operation modes of bioelectrochemical systems**.

**Oxygen level**	**O_2_**	**Aeration mode**	**Dissolved oxygen/ppb**
I	+	Headspace, passive (vent filter open)	35–50
II	++	Headspace, active for 48 h, then passive	35–50
III	+++	Medium, active for 48 h, then passive	1000 (active), 70 (passive)
III-	++(+)	Medium, active for 48 h, then no aeration	1000 (active), <15 (no air)

### Analytical methods

### Analysis of bacterial growth

Optical density at 600 nm and pH were monitored every 24 h for shake flask and bioelectrochemical experiments. In addition, total cell dry weight from each reactor was measured at the end of each experiment to account for biofilm formation. The total reactor volume was centrifuged, the pellets were dehydrated at 100°C overnight and dry cell weight was determined.

### Phenazine analysis

For a quick estimation of phenazine production, the concentration of oxidized pyocyanin was determined spectrophotometrically at 691 nm after vigorously vortexing the culture supernatant for 30 s. The concentrations of pyocyanin were calculated via Lambert–Beer's law using an extinction coefficient of 4.31 mM^−1^cm^−1^ for pyocyanin (Filloux and Ramos, 2014).

Quantitative phenazine analysis was performed via reversed phase high performance liquid chromatography RP-HPLC (Prominence UFLC, Shimadzu) using a C18 column (C18 Nucleodur ec, Macherey Nagel, Germany), equipped with a photo diode array UV/VIS detector (SPD-M20A, Shimadzu), which enabled detection and quantification of the phenazines at the following wavelengths: pyocyanin-319 nm, phenazine-1-carboxylic acid-366 nm, 1-hydroxyphenazine-257 nm (Mavrodi et al., 2001). Separation was achieved with a gradient of 25 mM ammonium acetate (eluent A) and acetonitrile (eluent B, LC-MS grade, Carl Roth GmbH, Germany) as eluents (with eluent A at 20% for 5 min, 80% for 10 min, and again 20% for 5 min) at a flow rate of 0.35 ml/min and a column temperature of 20°C. Phenazine peak areas were correlated to standard curves obtained by measuring pure commercial phenazines: PYO (Sigma Aldrich), PCA (Princeton BioMolecular Research Inc., New Jersey, USA), 1-OHPHZ (TCI Europe N.V., Belgium).

### Analysis of central metabolites

Glucose and secreted metabolites were analyzed via HPLC (Ultimate 3000 System, Dionex) with a 300 × 8.0 mm polystyrol-divinylbenzol copolymer (PS-DVB) separation column (CS-Chromatographie), a UV/VIS detector (Ultimate 3000 UV/VIS detector, Dionex) at 210 nm and a refractory index (RI) detector (RI-101, Shodex) at 35°C. Elution was achieved isocratically with 5 mM sulfuric acid at a flow rate of 0.6 ml min^−1^ at 75°C.

### Analysis of polyhydroxyalkanoates (PHAs)

Quantitative composition of the PHAs was determined by gas chromatography (Trace GC Ultra, Thermo Scientific) coupled to a mass spectrum analysis (ISQ, Thermo Scientific) of the extracted polyester. The extraction of the PHAs from dried cells was performed by methanolysis using (per 10 mg CDW) 2 mL chloroform and 2 mL methanol containing 15% sulfuric acid and 0.5 mg/mL 3-methylbenzoic acid as internal standard and then incubated at 100°C for 4 h (de Eugenio et al., 2010). Derivatization of the samples was performed using N-methyl-N-(trimethylsilyl) trifluoroacetamide (MSTFA, Macherey-Nagel, Düren) at 85°C for 1 h. For analysis, 1 μL of derivatized sample was injected by a split-splitless injector at a 1:50 split ratio into the gas chromatograph with a 1,4-bis(dimethylsiloxy)phenylene dimethyl polysiloxane separation column (30 m with 0.25 mm inner diameter, film thickness 0.25, Rxi-5Sil MS, Restek) at a flow rate of 0.9 mL/min with Helium as inert carrier gas. Electron ionization (EI) mass spectra were recorded in full scan mode (m/z 40–550). PHA monomers were identified by their specific mass spectra. The most commonly produced *P. putida* PHAs, 3-hydroxydecanoic acid and 3-hydroxydodecanoic acid, were commercially obtained as standards for sample quantification (Abcam plc, UK). The standards were treated like all other samples.

### Energy/charge balance calculations

For a global overview of the distribution of initial reducing equivalents as charge Q (provided as glucose input), an energy balance according to the following equation was calculated using Faraday's law (*Q* = *z* × *n* × *F*; with *z*, number of electrons per molecule; *n*, moles; *F*, Faraday constant):

$$\begin{array}{l} Q_{glucose\;input}=\;Q_{glucose\;left}+Q_{biomass}+Q_{metabolites}+Q_{PHAs}\\ \;+Q_{phenazines}+Q_{anode}+Q_{unaccounted} \end{array}$$

For the charge calculation of biomass based on the final cell dry weight, an elemental composition (as wt%) of biomass for *P. putida* KT2440 of 48.8% carbon, 6.2% hydrogen, 26.4% oxygen, 15.2% nitrogen, 2.7% phosphor, and 0.7% sulfur was assumed (van Duuren et al., 2013) leading to the following empirical molar sum formula for 1 mole of biomass: CH_1.52_O_0.41_N_0.27_P_0.02_S_0.054_ and a molecular mass of 24.6 g/mole.

Collected anodic charge was calculated via the integral of the recorded current over time, while charge for all other terms was calculated from the obtained quantitative analytical data.

## Results

### *P. putida* pPhz strain construction and evaluation

**Figure 1:**
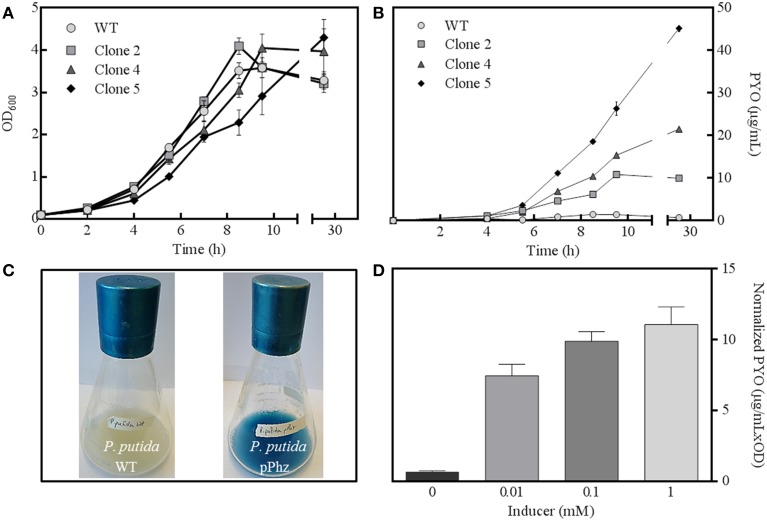
***P. putida* pPhz strain characterization. (A)** Optical density at 600 nm for three successful expression clones and the *P. putida* reference strain over time (all error bars indicate the standard deviation from triplicate shake flasks); **(B)** Estimation of phenazine synthesis by measuring PYO absorbance at 691 nm for cultures of Figure 1A; **(C)** Photographs of *P. putida* KT2440 wt (left) and *P. putida* pPhz (=clone 5, right) shake flask cultures showing phenazine synthesis; **(D)** Phenazine synthesis (via PYO absorbance at 691 nm) in dependency of salicylic acid inducer concentration (triplicate experiments).

The phenazine biosynthesis genes *phzA1-G1*, *phzM*, and *phzS* from *P. aeruginosa* were successfully cloned into the salicylate-inducible vectors pBNT and pJNN under the control of the salicylate-inducible promoter NagR/pNagAa to result in pBNTphzA-G and pJNNphzMS (Figures S1A–C). Five confirmed clones were subjected to replicate growth experiments in fully aerobic shake flasks to quantitatively examine growth behavior and phenazine production (Figures 1A,B). Two of the five clones (clones 1 and 3) only showed slight phenazine production over the unmodified reference strain (data not shown). For the other clones (2, 4, and 5), continuous phenazine synthesis with growth was observed to a maximum of 34 ± 0.86 μg/mL pyocyanin for clone 5 (with 0.1 mM salicylic acid as inducer). While the growth rate of the strains decreased with increasing phenazine synthesis (0.58 h^−1^ for the wild type and clone 2 vs. 0.46 h^−1^ for the highest producer clone 5), comparable final optical densities were reached. Likely, this deceleration of growth corresponds to the metabolic burden of efficient expression of the nine phenazine synthesis genes, together with the maintenance of two compatible plasmids under antibiotic selection. A time independent repetition of this experiment with fresh cultures from glycerol stocks confirmed the order of performance for all clones. Therefore, all following experiments were conducted with clone 5, hereafter designated as strain *P. putida* pPhz (Figure 1C).

Next, the activity of the salicylate-inducible promotor NagR/*pNagAa*, which controlled gene expression on both phenazine synthesis vectors, was evaluated (Figure 1D). Zero induction delivered very little phenazine synthesis (<1 μg/mL PYO) indicating a fairly tight control of the NagR repressed promoter. At 0.01 mM induction with salicylate, PYO levels of 7.4 ± 0.83 μg mL^−1^ OD^−1^ were determined, which increased to 11.1 ± 1.48 μg mL^−1^ OD^−1^ at 1 mM salicylate. All induced *P. putida* pPhz cells grew slower with increasing phenazine production (μ = 0.52 h^−1^ for 0.01 mM, μ = 0.47 h^−1^ for 0.1 mM, μ = 0.38 h^−1^ for 1 mM; compared to μ = 0.61 h^−1^ for the reference control). We also investigated the time of induction in an early growth phase (~2.5 h after inoculation, OD_600_ ~0.4) compared to a mid-logarithmic growth phase (~3.5 h after inoculation, OD_600_ ~0.8) (data not shown). More phenazine was produced when the inducer was added in the early log phase (final PYO was 34 μg/mL compared to 24 μg/mL at later induction). Therefore, all further experiments were conducted with induction of gene expression ~2.5 h after strain inoculation with 1 mM salicylic acid.

### *P. putida* pPhz current production under oxygen-limited conditions

#### Aeration regimes

**Figure 2:**
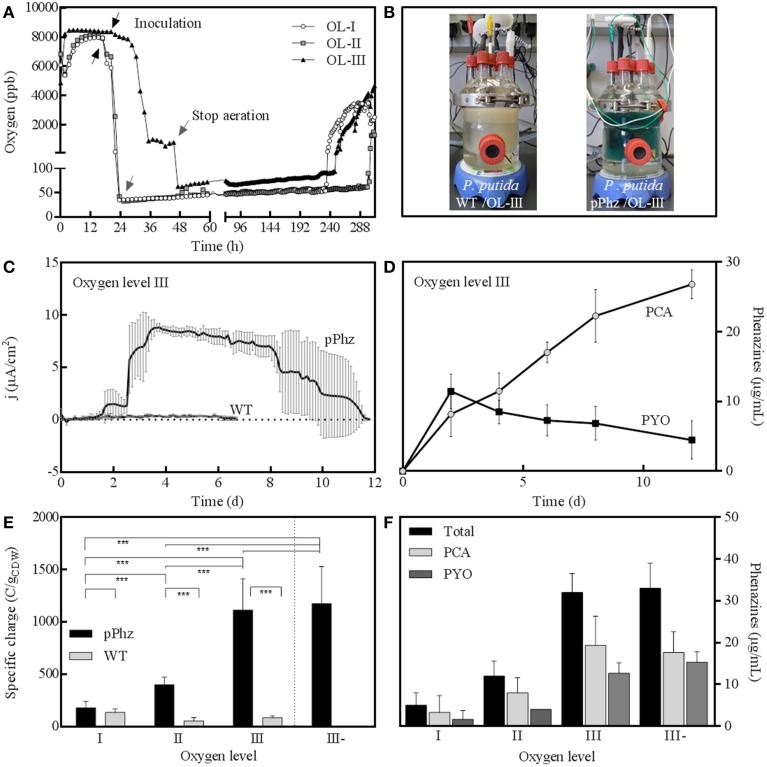
**Bioelectrochemical current generation of *P. putida* under oxygen limited conditions. (A)** Representative dissolved oxygen time profiles (in parts per billion) for oxygen levels I to III with OL-I: passive at 35–50 ppb O_2_; OL-II: active for 48 h, then passive at 35–50 ppm O_2_; OL-III: active for 48 h, then headspace at 70 ppb O_2_; **(B)** Photographic images of bioelectrochemical reactors with *P. putida* KT2440 wt (left) and *P. putida* pPhz with OL-III (right); **(C)** Anodic current density over time for *P. putida* pPhz compared to *P. putida* wt for OL-III, average of three replicates; **(D)** PYO and PCA concentrations for experiment in Figure 2D (measured via HPLC), average of three biological replicates; **(E)** Specific charge as accumulated anodic charge for the *P. putida* wt and pPhz strains normalized to the cell dry weight of the respective experiment for oxygen levels OL-I, OL-II, OL-III, and OL-III-. Statistical significance of difference was tested with an unpaired *t*-test between data linked by a bracket; with ^***^p < 0.05, ^**^p < 0.1, ^*^p < 0.2, no star: *p* > 0.2; **(F)** Phenazine concentration (PYO, PCA, and their sum) of *P. putida* pPhz at the different oxygen levels as in Figure 2E at their highest concentration during the experiment, respectively.

We examined the physiological response of the *P. putida* pPhz strain in a bioelectrochemical system with an electrode poised at 0.2 V vs. RE under different oxygen-limited conditions and compared it to the performance of the *P. putida* KT2440 reference strain. We applied four different oxygen limited operation regimes as listed in Table 1. For all conditions, representative dissolved oxygen concentrations are given in Figure 2A and Table 1. The increase in oxygen concentration for all reactors around days 10–12 (Figure 2A) corresponds with the death phase of the cultures (in accordance with a complete decline of current production; data not shown).

### Heterologous phenazine synthesis enables current generation in *P. putida* pPhz

The highest phenazine synthesis activity and the strongest bioelectrochemical current generation was observed for active media aeration as with OL-III and OL-III-. Figure 2B shows the intense color difference between the *P. putida* reference and the phenazine synthesizing *P. putida* pPhz at OL-III. Exemplary for all oxygen levels, the time-resolved current profile and the corresponding media concentration of PYO and PCA of *P. putida* pPhz compared to the *P. putida* reference for OL-III are shown (Figures 2C,D). While the reference produced hardly any electric current, the *P. putida* pPhz strain was able to sustain a current of ~8 μA/cm^2^ of anode surface area for >5 days.

The quantitative analysis of phenazines revealed a maximum of ~12 μg/mL PYO produced around day 2, where a first lower current plateau was observed (~1.5 μA/cm^2^) during the initial active aeration phase. Immediately after shifting the reactors to passive aeration, the current rose to its maximal value but PYO concentrations declined, while PCA continuously accumulated to a final concentration of 27 μg/mL. This is in agreement with the final phenazine synthesis steps from PCA to PYO directly involving an oxygen dependent monooxygenase. PCA hence could not efficiently be further converted to PYO during the passive aeration regime.

**Figure 3:**
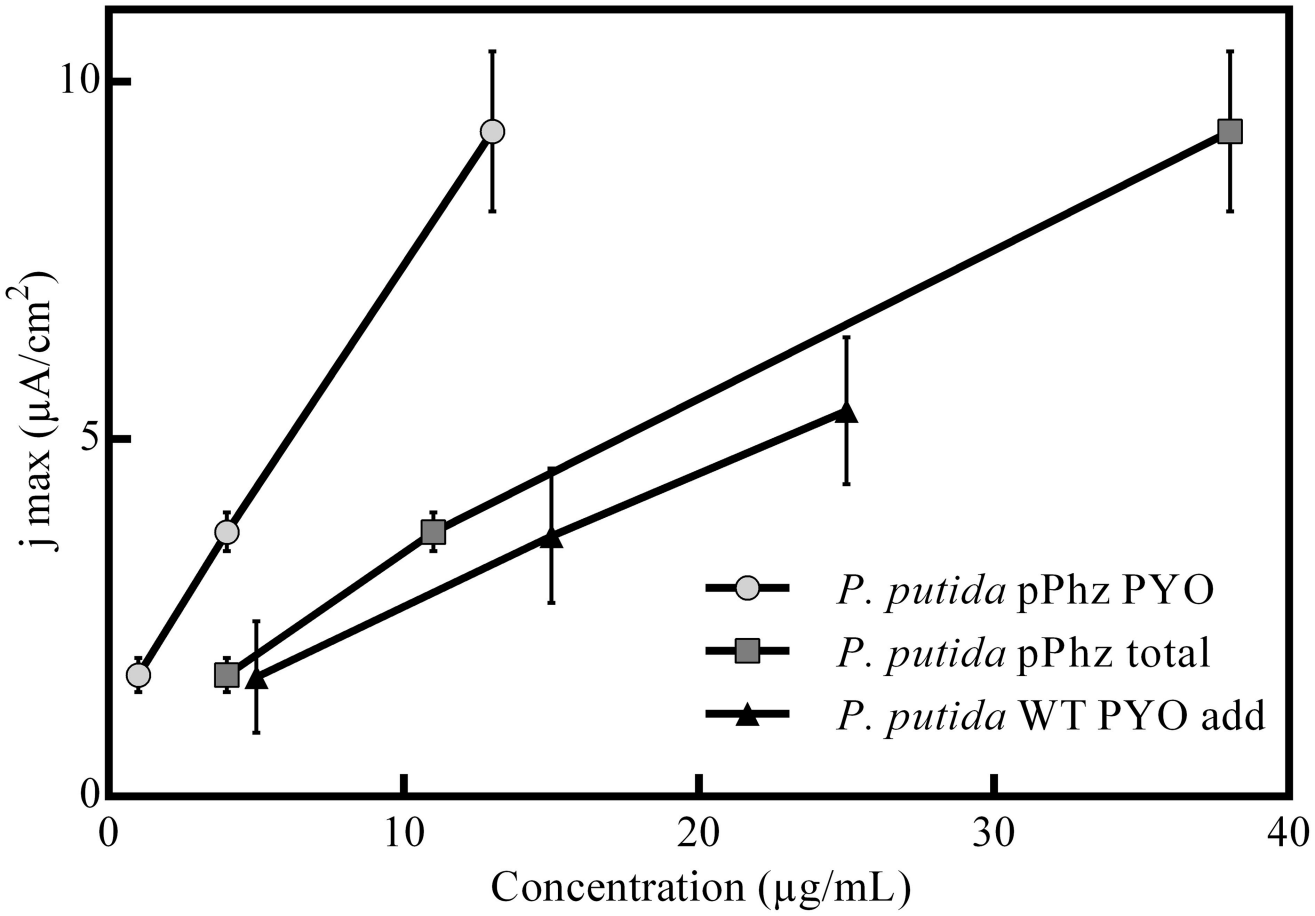
**Correlation between media phenazine concentration and recorded maximum anodic current density**. Shown are the engineered *P. putida* pPhz strain and the reference strain in a control experiment with the addition of commercial (natural) PYO at 5, 15, and 25 μg/mL. The linear correlation coefficients are R^2^-pPhz PYO: 0,9998, R^2^-pPhz total: 0,9964, R^2^-WT PYO add: 0,9988.

A compilation of bioelectrochemical data for the *P. putida* reference vs. pPhz strain under all conditions is shown in Table 2 and Figures 2E,F. Even the non-phenazine producing *P. putida* KT2440 wt has some limited ability for current generation at a poised anode, most pronounced under the most stringent oxygen-limited condition (OL-I). In contrast, the ability to interact with the anode and generate an electric current increased with the availability of initial oxygen for *P. putida* pPhz (Figure 2E). At OL-I, the KT2440 wt strain generated about 70% of the average current and charge of the new pPhz strain, while this shifted to only 10% of the average current and 7% of the charge for OL-III). The increase in current with oxygen levels for *P. putida* pPhz directly corresponds to an increase in synthesized phenazines with oxygen availability (Figure 2F). This, however, is not caused by an increase in biomass during initial growth (instead biomass decreases—Table 2). Figure 3 shows a direct linear correlation between the measured current density and the phenazine concentrations.

**Table 2:** **Data summary for oxygen limited bioelectrochemical experiments**.

**O_2_**	**Strain**	**Time (d)**	**End pH**	**Final CDW (g/L)**	**Biomass yield **Y_x/s_** (g_**CDW**_/g_**Gluc**_)**	**j max (μA/cm^2^)**	**j avg. (μA/cm^2^)**	**Collected charge (C)**
I	WT (no anode)	10	5.93 ± 0.22	0.90 ± 0.16	0.20 ± 0.018	–	–	–
I	WT	12	5.62 ± 0.05	1.85 ± 0.11	0.41 ± 0.01	1.5 ± 0.76	0.9 ± 0.15	144 ± 40
I	pPhz	12	5.79 ± 0.09	2.20 ± 0.19	0.44 ± 0.07	1.7 ± 0.24	1.3 ± 0.37	211 ± 60
II	WT	13	5.92 ± 0.02	1.57 ± 0.38	0.33 ± 0.07	0.4 ± 0.30	0.3 ± 0.01	46 ± 25
II	pPhz	14	5.79 ± 0.10	1.60 ± 0.08	0.39 ± 0.03	3.7 ± 0.27	2.2 ± 0.19	417 ± 41
III	WT	9.5	5.60 ± 0.21	1.15 ± 0.25	0.23 ± 0.03	0.8 ± 0.09	0.6 ± 0.23	58 ± 43
III	pPhz	10	6.01 ± 0.07	1.50 ± 0.39	0.35 ± 0.08	9.3 ± 1.12	5.1 ± 2.24	819 ± 149
III-	pPhz	16	5.96 ± 0.15	1.40 ± 0.40	0.36 ± 0.07	12 ± 0.64	4.3 ± 1.78	977 ± 398

Statistical evaluation of the electrochemical data (Figure 2E, level of significance is indicated by stars) showed a highly significant difference in current generation for *P. putida* pPhz vs. reference for OL-II and OL-III (*p* < 0.05) and for the effect of the initially provided oxygen for *P. putida* pPhz except for the test of OL-III vs. OL-III-. Here, no statistically significant difference was found, but also phenazine levels were similar (Figure 2F). Standard deviations of accumulated charges were comparatively large because the decline in current production toward the end of the experiment set in at different time points for the three replicates.

We also performed experiments to evaluate the tolerance of *P. putida* KT2440 toward artificial PYO addition under passive headspace aeration conditions (OL-I). It was confirmed that the current production increased when comparable concentrations of PYO (from 5 to 25 μg/mL) were artificially added. However, the unmodified *P. putida* KT2440 with added pyocyanin showed a lower anodic current generation than the engineered *P. putida* pPhz strain at similar pyocyanin concentrations (Figure 3).

### Metabolic effects of oxygen limitation and anodic electron discharge in *P. putida* pPhz

#### *P. putida* growth and biomass formation

We observed discrepancy when evaluating growth during experiments via optical density compared to the total dry cell weight measured at the end of each experimental run (growth profiles of selected experiments in Figure S2A). This is partially due to biofilm formation in the reactors, but might also be indicative of different cell morphologies under oxygen-limited conditions. We therefore based all final growth evaluations on the measured dry cell weight. As expected, the growth rate and biomass yield per consumed substrate (Y_x/s_) was low for the *P. putida* KT2440 reference strain under oxygen limitation (OL-I) when no electrode was present (Table 2). Under the same aeration condition, the biomass yield Y_x/s_ doubled when a poised electrode was available as alternative electron acceptor (Y_x/s_: 0.41 vs. 0.2 g_CDW_/g_glc_ with an electrode compared to without electrode, respectively). With increasing initial availability of oxygen for OL-II and OL-III, Y_x/s_ decreased to 0.23 g_CDW_/g_glc_ for the reference strain (OL-III, Table 2). For *P. putida* pPhz, we observed the highest total biomass and biomass yield for the lowest oxygen level (OL-1, Table 2). Total biomass and biomass yield for all other oxygen levels were fairly similar. However, for all oxygen-limited bioelectrochemical experiments Y_x/s_ was much lower than is normally observed for aerated *P. putida* KT2440 cultivations on glucose (Y_x/s_ = 0.57 g_CDW_/g_glc_)(Blank et al., 2008).

#### Side products of glucose metabolism

**Figure 4:**
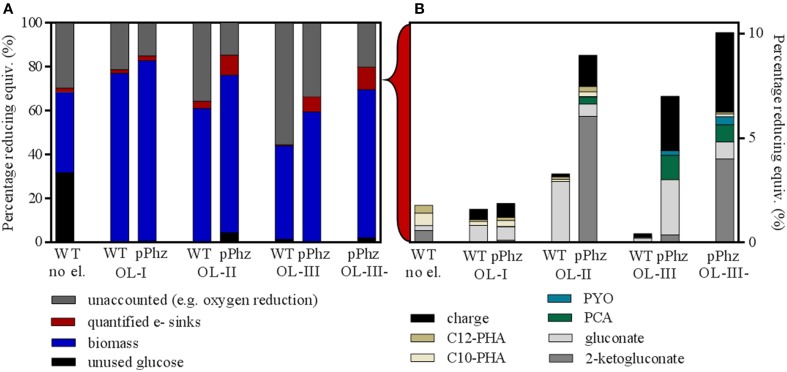
**Differential distribution of input reducing equivalents for the *P. putida* reference and pPhz strains at different oxygen levels. (A)** Distribution of input reducing equivalents (as percentage of glucose input) to unutilized glucose, biomass, other quantified electron sinks and unaccounted electron sinks such as oxygen respiration. The first bar represents the reference strain at OL-I without an electrode. **(B)** Break-up of input energy distribution (in reducing equivalents) to other quantified electron or energy sinks: residual metabolites gluconate and 2-ketogluconate; synthesized pyocyanin and phenazine-1-carboxylic acid; internal C10 and C12 PHA storage molecules; and collected electric charge. For all experiments, the balance was calculated based on end-of-experiment composition of biomass and media. All calculations are based on triplicate experiments, except for the reference at OL-I and OL-III, which were duplicates.

*P. putida* has several biochemical pathways for the uptake of glucose for energy generation (Ebert et al., 2011). Besides the uptake via the common direct glucose transporter, glucose can also be oxidized in the periplasm to gluconate and further to 2-ketogluconate, releasing up to four electrons as reducing equivalents. It is known from related *P. aeruginosa* that oxygen limitation can shift the glucose metabolism from the periplasmic direct oxidative conversion to gluconate and 2-ketogluconate to the intracellular phosphorylative route of glucose consumption (Mitchell and Dawes, 1982). During our experiments, the concentrations and ratio of gluconate to 2-ketogluconate in the culture supernatant varied over time. Table 3 reports the maximal concentrations observed, however, for a final energetic balancing of reactor operations (Figure 4) we used end point concentrations. For the *P. putida* KT2440 reference, we observed generally low gluconate and 2-ketogluconate concentrations with a maximal accumulation for OL-II. Comparably low levels were observed for the *P. putida* pPhz strain at OL-I, but it significantly increased over the reference strain for OL-II and OL-III, corresponding with more phenazine synthesis and electrode availability under these conditions. The highest accumulation of 2-ketogluconate was observed during the anaerobic, high current producing phase of OL-III- (Table 3 and Figure S2B). This could indicate that a partial glucose oxidation to 2-ketogluconate might be beneficially linked to phenazine electron discharge to the anode when no oxygen is available.

**Table 3:** **Summary of metabolic side product formation for different oxygen levels**.

**O_2_**	**Strain**	**Gluconate (mM)**	**2-Ketogluconate (mM)**	**Acetate (mM)**	**PHAs (C10 + C12)^a^ (μg/mgCDW)**
I	WT (no electrode)	0.37 ± 0.18	0.18 ± 0.08	0.75 ± 1.30	15.2 ± 0.4
I	WT	0.58 ± 0.13	0.09 ± 0.03	0	3.3 ± 0.8
I	pPhz	0.55 ± 0.11	0.06 ± 0.08	0.17 ± 0.05	4.8 ± 2.5
II	WT	0.83 ± 0.13	0.29 ± 0.28	1.22 ± 0.12	7.3 ± 5.3
II	pPhz	1.74 ± 0.13	1.77 ± 0.20	0.47 ± 0.50	5.8 ± 1.5
III	WT	0.14 ± 0.03	0.10 ± 0.01	0	3.7 ± 3.5
III	pPhz	2.86 ± 2.67	0.48 ± 0.52	0.28 ± 0.49	2.9 ± 0.5
III-	pPhz	0.42 ± 0.18	3.80 ± 2.16	0	1.0 ± 2.2

a *sum of poly-3-hydroxy-decanoate, C10, and poly-3-hydroxy-dodecanoate, C12*.

Our HPLC analysis of the culture supernatant further showed traces of acetate (<2 mM), maybe as result of unknown side reactions under our oxygen-limited growth conditions (Table 3).

Another common *P. putida* side product, especially under nutrient limiting conditions, are polymeric accumulations of polyhydroxyalkanoates as intracellular energy storage molecules (Escapa et al., 2012). Therefore, we analyzed the collected cell dry weight for accumulated PHAs at the end of each experiment (Table 3 shows the sum of the two most common PHAs of *P. putida* KT2440: poly-3-hydroxy-decanoate and poly-3-hydroxy-dodecanoate). Generally, we found less PHA accumulation in bioelectrochemical experiments compared to our non-electrode control of *P. putida* KT2440. In dependency of the oxygen levels, no clear trends can be deduced, even though there is a tendency for less PHA accumulation with higher current generation for the pPhz strain.

#### Overall distribution of glucose reducing equivalents

Figure 4 shows an overall comparison of the distribution of the energy stored in input glucose to biomass, electric current, and metabolic side products for all aeration conditions. For this, all measurements were converted to charge equivalents. Under oxygen-limited conditions with an electrode, glucose was always almost completely consumed (>95%) by the end of the experiment (Figure 4A). However, for the *P. putida* reference without an anode about 32% of the glucose remained unused. For all experiments, the majority of the input reducing equivalents was recovered as biomass (based on the assumed composition as stated in Section Energy/Charge balance calculations). A fraction between 15 and 55% of the input reducing equivalents could not be quantitatively accounted for. This fraction increased with the provided oxygen during the initial growth phase and therefore, most likely is attributed mainly to oxygen respiration. All other quantified electron sinks only accumulated to a small fraction of the input reducing equivalents (detailed in Figure 4B).

In comparison to the reference, the *P. putida* pPhz strain produced slightly more biomass at each oxygen level, the amount of unaccounted for reducing equivalents was lower, and the total amount of quantifiable electron sinks increased. Even though more oxygen was provided during the initial growth phase, the increased production of phenazines resulted overall in a pronounced electron discharge to the anode rather than to oxygen. Overall, the fraction of reducing equivalents discharged to the anode rose from 0.7 to 2.6% from OL-I to OL-III (Figure 4B). The increase in synthesized phenazines accounts for up to 1.4% of the input reducing equivalents for OL-III. One group of side products of growing significance in the pPhz strain with increasing oxygen availability are gluconate and 2-ketogluconate resulting from the periplasmic partial oxidation of glucose.

In comparison, the distribution of reducing equivalents for OL-III, which included a passively aerated headspace after initial media aeration, and OL-III-, which was completely cut off from aeration after the initial media aeration, are fairly similar for biomass and accumulated charge. However, the glucose oxidation product 2-ketogluconate further increased for OL-III-.

## Discussion

### Heterologous phenazine synthesis enables anodic electron discharge by *P. putida* KT2440

For the first time, we here show the molecular engineering of a non- or barely electroactive biotechnologically relevant microorganism—*P. putida* KT2440—to synthesize and utilize soluble redox mediators for metabolic electron discharge to an anode. The heterologous expression of the nine phenazine synthesis genes from *P. aeruginosa* in *P. putida* KT2440 resulted in the production of almost exclusively pyocyanin and phenazine-1-carboxylic acid, whereby PYO was only synthesized when oxygen was still available during the initial growth phase. During subsequent passive headspace aeration, PYO concentrations slowly subsided over time while PCA was further synthesized and accumulated (Figure 2). Thereby, the total produced phenazine concentrations were in the upper range of phenazine concentrations found in metabolically stimulated *P. aeruginosa* [30–40 μg/mL for OL-III and –III- for *P. putida* pPhz vs. 10–25 μg/mL for *P. aeruginosa* PA14 (Venkataraman et al., 2010, 2011)].

With increasing availability of oxygen during initial growth, more phenazines were produced. Phenazine concentrations correlated well (linear relationship, Figure 3) with the increasing ability to generate an anodic current (both if total phenazines or just PYO is considered). This is in accordance with the findings for *P. aeruginosa*, where BES have been proposed as direct *in situ* quantification tool for the production of phenazines (Venkataraman et al., 2010).

Experiments with addition of PYO to the reference strain showed lower electrochemical activity than our engineered strain (Figure 3). In the *P. putida* pPhz strain, PYO concentrations were maximal ~15 μg/mL (in contrast to addition up to 25 μg/mL in the control experiment), while PCA was the dominating phenazine during the current production phase. It is unclear, yet, if the difference in current production between externally added PYO and the pPhz strain is because strain pPhz produces a mixture of phenazines, which all participate in electron transfer, or if intracellularly produced pyocyanin might be more effective as a metabolic electron acceptor. This question will be targeted in subsequent experiments. A general toxic effect of phenazines at the produced levels can be excluded from our experiments, since final optical densities under fully aerobic conditions (Figure 1A; where phenazines should be most toxic due to formation of oxygen radicals) were similar for the reference and the phenazine producing strains. Decreasing growth rates with increasing ability to produce phenazines more likely are caused by the metabolic burden to efficiently synthesize the nine heterologous phenazine synthesis proteins and the maintenance of the two plasmids under antibiotic selection.

One great advantage of heterologous phenazine production in *P. putida* over native production in *P. aeruginosa* (besides working in a non-pathogenic host) is that in *P. putida* production is only dependent on the defined induction of gene expression with salicylic acid and the availability of oxygen (for increased biomass and PYO synthesis). Oxygen influenced the ratio of PYO to PCA because the conversion from PCA to PYO is catalyzed by an oxygen-dependent monooxygenase (PhzS). In the future, a modified engineered strain only expressing PhzA-G to synthesize PCA (but not PYO) should be investigated, since PCA was the major phenazine during current production (Figures 2C,D) and under the most stringent oxygen levels (OL-III and OL-III-). Likely in such a strain, phenazine synthesis could be fully controllable via defined induction of gene expression independent of the cultivation conditions used. In contrast, phenazine synthesis by *P. aeruginosa* is controlled by the complex quorum sensing regulatory network, which not only responds to population dynamics but is also strongly influenced by many—often hardly understood—environmental factors, such as nutrient source or oxygen availability. Thus, from a biotechnological standpoint, the controllable, heterologous production of phenazine redox mediators in *P. putida* provides a great chance for targeted redox homeostasis.

### Anodic redox balancing with *P. putida* influences energy metabolism and growth

We quantified the distribution of provided reducing equivalents (from glucose input) to possible electron sinks to investigate the metabolic effect of phenazine availability for anodic electron discharge (Figure 4). When comparing the *P. putida* reference strain with passive headspace aeration (OL-I) with and without an anode, a change in metabolic activity is visible. Without the electrode almost 32% of the provided glucose remained unused and only half as much biomass was formed compared to when an anode was present. Thus, just the simple presence of an anode as electron acceptor or maybe the influence of the redox potential control in the bioelectrochemical experiment seemed to cause a dramatic shift in substrate utilization and cell growth. Further, an increase in oxygen availability during the initial growth phase (OL-II or OL-III) decreased the biomass yield on glucose (Table 2 and Figure 4A). This effect requires further investigation.

Analyzing the quantifiable electron sinks, it is notable that the reference strain without anode accumulated the most internal PHAs (C10 + C12) over all tested strains and conditions. This is likely because of the limited growth ability under this reference condition. An externally available anode might reduce the necessity to store away reducing equivalents internally to some degree. When an anode is present, the reference strain shows some limited ability to discharge reducing equivalents, but this ability is greater with less oxygen available (higher current for OL-I than for OL-II or OL-III). It is unclear how unmodified *P. putida* KT2440 utilizes the electrode for discharge. But it is possible that undefined redox compounds act as electron mediators to specifically discharge electrons, or that the controlled oxidized redox conditions (+0.2 V vs. RE ~ +0.4 V vs. SHE) in general sustain an oxidized growth medium environment that might act as a non-specific electron acceptor (in the sense of a redox “buffer”). This speculation is supported by the fact that the influence of the anode (participation as electron sink) becomes weaker with increasing availability of oxygen, which then can keep the media environment in its more oxidized state.

With increasing oxygen levels, we found more gluconate and 2-ketogluconate for the pPhz strain. It is known from studies of *P. aeruginosa* that glucose metabolism shifts away from periplasmic direct oxidation under oxygen limitation (Mitchell and Dawes, 1982). Therefore, by increasing initial oxygen levels and thereby increasing anode availability, we seemed to enhance utilization of this oxidative route for *P. putida*. In follow-up experiments it should be investigated if the hereby released reducing equivalents can directly feed into the phenazine-based electron discharge to the anode. Since 2-ketogluconate is an industrially attractive biochemical, the coupling of periplasmic glucose oxidation to 2-ketogluconate with anaerobic electron discharge to an anode could present an attractive and economic way for biotechnological 2-ketogluconate synthesis.

We were able to show that after an initial provision of oxygen during the first 48 h of growth for experiments done under OL-III and OL-III- conditions, a fairly stable anaerobic physiological state was sustained for 2 weeks, which did not require any further aeration to keep the cells metabolically active [indicated by active electron discharge to the anode (Figure 2C, Table 2), glucose consumption and product formation (Figure S2)]. This result should be taken into consideration when engineering future phenazine producing *P. putida* strains for anaerobic biocatalysis as outlined in the next section.

Previous research work to engineer anaerobic metabolism in *P. putida* KT2440 via nitrate respiration (Steen et al., 2013) or ethanol fermentation (Nikel and de Lorenzo, 2013) also yielded metabolically active cells during anaerobic cultivation. However, these cells were not able to grow. At this point, it is not clear from our experiments, how growth was influenced after the initial 48 h of aeration, since the optical density measurements (Figure S2) were not always reliable due to strong biofilm formation. Thus, the OD measurements, which indicate slow growth for OL-III until day 6 after inoculation and even slower growth for OL-III- until day 9, should be regarded with care and further experiments are required.

### Next steps for combining oxygen-limited growth of *P. putida* with biocatalysis

**Figure 5:**
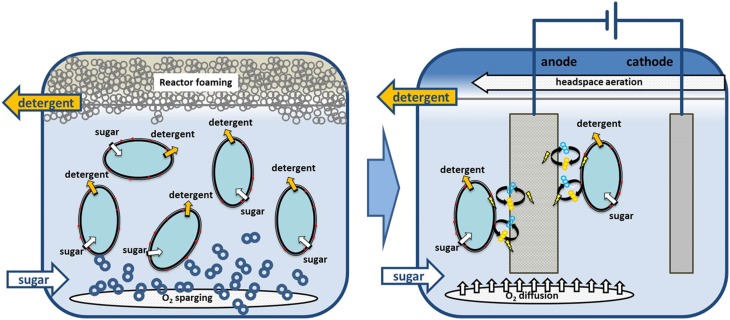
**Example process scenario for switching *P. putida* from obligate aerobic to non-foaming, oxygen limited biodetergent production**. We anticipate combining biodetergent (e.g., rhamnolipid) production with phenazine-based redox balancing at an anode. Required oxygen for initial biomass growth or phenazine synthesis could then be supplied via passive diffusion to prevent bioreactor foaming.

With our phenazine producing *P. putida* pPhz strain, oxygen-limited growth and production become feasible through the discharge of surplus reducing equivalents to an anode. Oxygen-limited applications with *P. putida* are highly desirable for the bioproduction of amphiphilic compounds such as rhamnolipids or hydrophobic compounds, which require a second non-polar phase for *in-situ* product removal. Under such conditions, high-level aeration, which is currently required for *P. putida* biocatalysis, frequently leads to reactor foaming, making large scale applications challenging (Küpper et al., 2013). In a next important step, the true capacity of phenazine-based redox balancing under bioproduction conditions needs to be evaluated. We anticipate combining our well established heterologous production of rhamnolipids (Wittgens et al., 2011) (originating also from *P. aeruginosa*) with oxygen-limited phenazine redox balancing in one *P. putida* host strain. Figure 5 illustrates our concept of moving from an aerated to an oxygen-limited production process for rhamnolipids, where oxygen is only supplied in the headspace and/or via passive diffusion to prevent reactor foaming.

In this combined oxygen-limited bioproduction strain, we will be able to quantitatively assess the efficiency of redox balancing via phenazines and an anode. We observed in this initial proof-of-principle study that after initial low oxygen growth for 48 h, stable metabolic activity and redox balancing of the cultures sustained for about 14 days under very stringent oxygen conditions. This could provide multiple scenarios for dynamic bioprocess steering between aerobic biocatalyst growth phases and efficient oxygen-limited or even oxygen-free production phases. However, the energetic requirements for any biocatalytic process will likely influence or change the cells capacity to sustain anaerobic metabolism and therefore need to be evaluated in a consolidated approach.

Another important outcome of this work is the observation that PYO is mainly produced during the first 48 when some oxygen is still present for its final synthesis step. During the remainder of the experiments, PYO concentrations declined while PCA was further synthesized and represented the main phenazine responsible for sustaining the anodic current production (Figure 2D). Thus, a new version of our *P. putida* pPhz strain should be investigated, which only expresses *phzA-G* to synthesize PCA. Omitting plasmid pJNNphzMS could limit the metabolic burden on the *P. putida* strain (two genes less to express) and reduce the antibiotic stress (requirement of only one of the two antibiotics). Further, it could possibly open the opportunity to work fully anaerobic if PCA-based redox balancing could fulfill the required metabolic energy conservation for ATP synthesis. At this stage, it is unclear if, and how efficiently, phenazine-based electron discharge participates in cell energy conservation and whether or not an intermittent aerobic phase is still required for cell growth. Thus, both from a biotechnological as well as a basic science standpoint this initial proof-of-principle work toward phenazine-based redox balancing in *P. putida* provides a fruitful platform for extended future research.

## Author contributions

SS performed all characterization experiments, analyzed the data, prepared figures and edited the manuscript. SN performed the molecular engineering experiments, prepared figures and edited the manuscript. NW provided guidance on *P. putida* molecular engineering, discussed the data and critically revised the manuscript. LB and MR conceived of the study and obtained funding. LB provided guidance on *P. putida* biotechnology and edited the manuscript. MR advised on all experiments, provided guidance on bioelectrochemical testing, analyzed and discussed data, and wrote the manuscript.

### Conflict of interest statement

The authors declare that the research was conducted in the absence of any commercial or financial relationships that could be construed as a potential conflict of interest.
