# Supplementary material for: Engineering mediator-based electroactivity in the obligate aerobic bacterium Pseudomonas putida KT2440
Source: Front Microbiol. 2015 Apr 10;6:284. doi: 10.3389/fmicb.2015.00284 (PMC4392322; doi:10.3389/fmicb.2015.00284)
Supplement: Supplementary file 1 [file DataSheet1.DOCX]

***Supplementary Material***

**Engineering mediator-based electroactivity in the obligate aerobic bacterium *Pseudomonas putida* KT2440**

**Simone Schmitz, Salome Nies, Nick Wierckx, Lars M. Blank, Miriam A. Rosenbaum***

Institute of Applied Microbiology, Aachen Biology and Biotechnology, RWTH Aachen University, Aachen, Germany

*** Correspondence:** Prof. Miriam A. Rosenbaum, Institute of Applied Microbiology, RWTH Aachen University, Worringerweg 1, 52074 Aachen, Germany.

miriam.rosenbaum@rwth-aachen.de

1. **Supplementary Figures and Tables**

This supplementary document contains supporting data for the molecular engineering of our new phenazine producing strain *P. putida* pPhz (Tables S1 and S2 as well as Figure S1). Further, the detailed metabolic data for experiments at OL-III and OL-III- are presented in Figure S2 to support the discussion of our results.

## Supplementary Tables

**Supplementary Table S1.** Bacterial strains and plasmids used in this study

| **Strain or plasmid** | **Relevant characteristics^a^** | **Source or Ref.** |
| --- | --- | --- |
| Strains |  |  |
| *P. aeruginosa* PAO1 | wild type, genomic DNA | DSM19880 |
| *E.coli* DH5α | *fhuA*2 *lac*(del)U169 *phoA glnV*44 Φ80' *lacZ*(del)M15 *gyrA*96 *recA*1 *relA*1 *endA*1 *thi*-1 *hsdR*17 | New England Biolabs |
| *E. coli* DH5α pBNTphzA-G | *E.coli DH5α* containing plasmid pBNTphzA-G | This study |
| *E. coli* DH5α pJNNphzMS | *E.coli DH5α* containing plasmid pJNNphzMS | This study |
| *P. putida* KT2440 | *P. putida* mt-2 wild type lacking megaplasmid ([Bagdasarian et al., 1981](#_ENREF_1)) | DSM6125 |
| *P. putida* pPhz | *P. putida* KT2440 containing plasmids pJNNphzMS and pBNTphzA-G | This study |
|  |  |  |
| Plasmids |  |  |
| pBNT mcs | Km^r^, expression vector containing the salicylate-inducible *nagR*/*pNagAa* promotor | ([Verhoef et al., 2010](#_ENREF_13)) |
| pJNN mcs(t) | Gm^r^, expression vector containing the salicylate-inducible *nagR/pNagAa* promotor | ([Wierckx et al., 2008](#_ENREF_14)) |
| pBNTphzA-G | Km^r^, derived from pBNT mcs, contains the operon *phzABCDEFG* under control of the *NagR/pNagAa* promotor | This study |
| pJNNphzMS | Gm^r^, derived from pJNN mcs(t), contains the *phzM* and *phzS* genes under control of the *NagR/pNagAa* promotor | This study |

^a^ Gm^r^, Km^r^, gentamycin, kanamycin resistance, respectively.

**Supplementary Table S2.** Primers used in this study

| **No.** | **Primer** | **Sequence 5’- ^a^** | **Function** |
| --- | --- | --- | --- |
| 1 | pJNN fwd | cggtaccgaattcctcgag*tctaga*caaccgttgaaagttccgatatgaataattc | pair for amplification of gene *phzM,* with overlapping regions for Gibson assembly, *Xba1* restriction site |
| 2 | phzM rev | tgttcgaatctcaggccctggcagcgac |  |
| 3 | phzS fwd | cagggcctgagattcgaacactcgagaaaaggaag | pair for amplification of gene *phzS,* with overlapping region for Gibson assembly |
| 4 | pJNN rev | gcccgacgtcgcatgctcctgctagcgtggccgttcca |  |
| 5 | pBNT phzA-G fwd | accgaattcctcgag*tctaga*ggaagcatcagcttagcaatcccgc | pair for amplification of operon *phzABCDEFG, Xba*1 restriction site |
| 6 | pBNT phzA-G rev | acgtcgcatgctcctttatccgccatgaaacgccg |  |
| 7 | pJNN Seq 1 | agaatcgcagccattcg | Sequencing primer for pJNN |
| 8 | pJNN Seq 2 | ccaacggcgatatctac |  |
| 9 | pJNN Seq 3 | agttcgaccgcttcctg |  |
| 10 | pBNT Seq 1 | gtcaacgcgaacatttcc | Sequencing primer for pBNT |
| 11 | pBNT Seq 2 | accaaaggccaggatcg |  |
| 12 | pBNT Seq 3 | atcctcaagggctatgc |  |
| 13 | pBNT Seq 4 | tgcagcgctacttcctac |  |
| 14 | pBNT Seq 5 | gaactgagcgaggaagg |  |
| 15 | pBNT Seq 6 | accatcgtgcgtcactc |  |
| 16 | pBNT Seq 7 | gcggtgttcttcgactg |  |

^a^ Underlined nucleotides refer to gene-specific region, italic style marks the restriction site

## Supplementary Figures


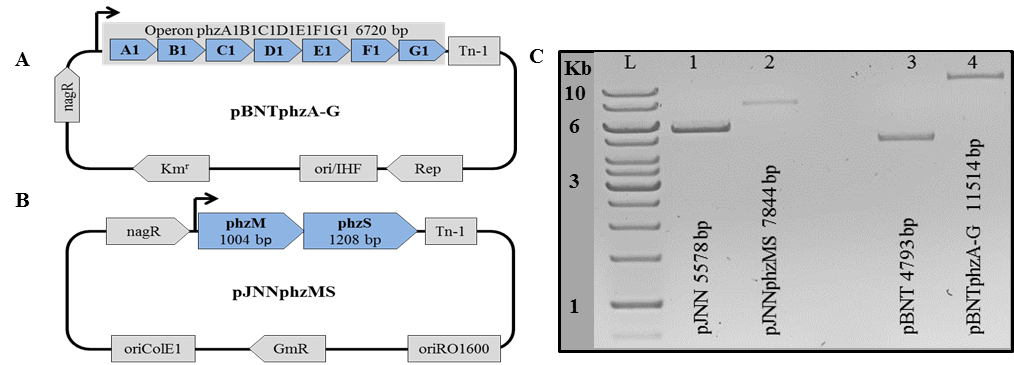


Supplementary Figure 1. *P. putida* pPhz strain construction. A) Plasmid map of pBNTphzA-G showing the *phzA1-G1* gene operon, the location of the NagR*/pNagAa* promotor system, the kanamycin resistance cassette and further plasmid features; B) Plasmid map of pJNNphzMS showing the genes *phzM* and *phzS* under control of the NagR*/pNagAa* promotor system, the gentamycin resistance cassette and further important plasmid features; C) DNA agarose gel (1 %) showing the linearized empty and assembled gene expression vectors at their appropriate molecular size.


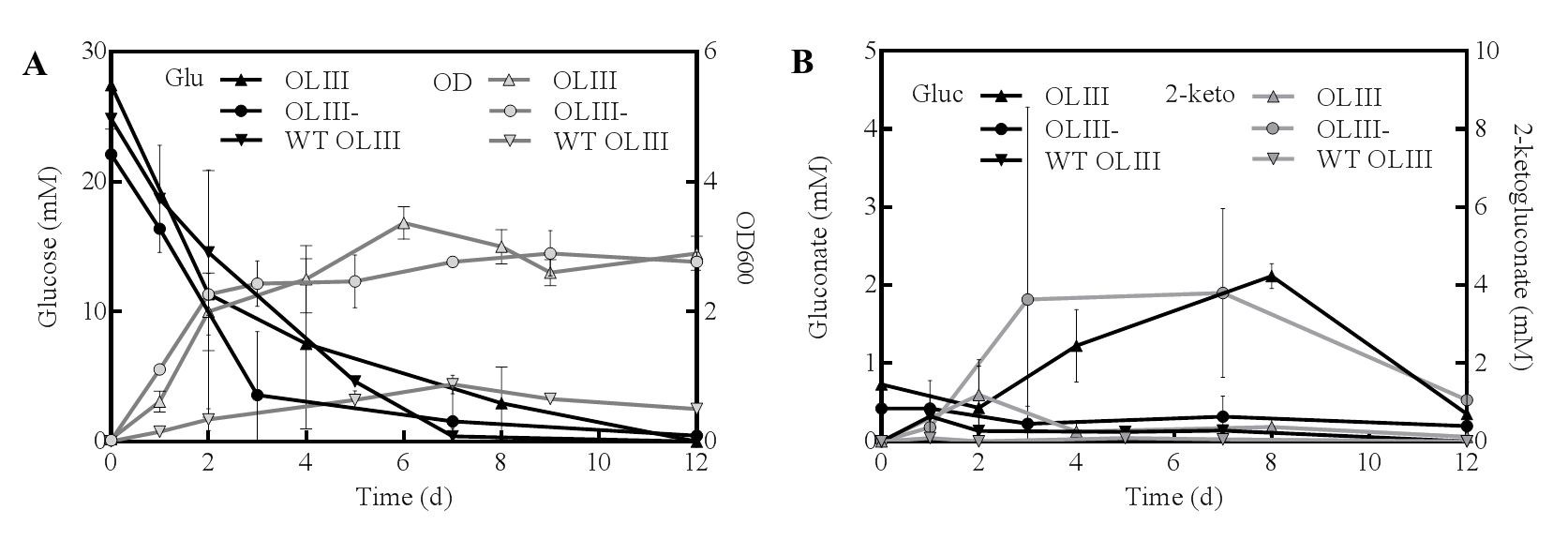


**Supplementary Figure 2. Metabolite profiles for OL-III and OL-III- experiments. A)** Glucose consumption and microbial growth as optical density at 600 nm for bioelectrochemical experiments with the *P. putida* pPhz strain at OL-III and OL-III- and the *P. putida* KT2440 wt at OL-III. Triplicate experiments, except duplicates for KT2440; **B)** Gluconate (left axis) and 2-ketoglutarate (right axis) formation during bioelectrochemical experiments with the *P. putida* pPhz strain at OL-III and OL-III- and the *P. putida* KT2440 wt at OL-III.
